# The Influence of Genetic Polymorphisms and Psychosocial Determinants on Suicidal Behaviors: A Case–Control Study of *CRHR1*, *NTRK2*, and *FKBP5*

**DOI:** 10.3390/ijms26168053

**Published:** 2025-08-20

**Authors:** Mihaela Elvira Cîmpianu, Emilian Onișan, Viviana Maria Sărac, Ioan Sărac, Mariana Ganea, Gligor Octavia, Ștefana Bâlici, Gheorghe Zsolt Nicula, Elena Maria Domșa, Teodora Cîmpianu, Sergiu Ionica Rusu, Horia George Coman, Mihaela Laura Vică Matei, Costel Vasile Siserman

**Affiliations:** 1 Department of Cellular and Molecular Biology, Iuliu Haṭieganu University of Medicine and Pharmacy, 400006 Cluj-Napoca, Romania; sbalici@umfcluj.ro (Ș.B.); gnicula@umfcluj.ro (G.Z.N.); mvica@umfcluj.ro (M.L.V.M.); 2 Department of Social Sciences, “1st December 1918” University of Alba Iulia, 510009 Alba Iulia, Romania; maria.domsa@uab.ro; 3 Legal Medicine Institute, 400006 Cluj-Napoca, Romania; cvsiserman@gmail.com; 4 Genetic Department, University of Life Sciences “King Mihai I” from Timisoara, 300645 Timisoara, Romania; ioansarac@usvt.ro; 5 Faculty of Pharmacy, “Victor Babeş” University of Medicine and Pharmacy, 2 Eftimie Murgu Square, 300041 Timisoara, Romania; saracvivianamaria@gmail.com; 6 Department of Pharmacy, Faculty of Medicine and Pharmacy, University of Oradea, 29 Nicolae Jiga Street, 410028 Oradea, Romania; mganea@uoradea.ro; 7 Department of Preclinical Disciplines, Faculty of Medicine and Pharmacy, University of Oradea, 10, 1 December Square, 410073 Oradea, Romania; octavia.gligor@uroadea.ro; 8 Faculty of Medicine, Iuliu Haṭieganu University of Medicine and Pharmacy, 400349 Cluj-Napoca, Romania; cimpianu.teodora@elearn.umfcluj.ro; 9 Faculty of Sociology and Social Work, “Babeș-Bolyai” University, 400084 Cluj-Napoca, Romania; sergiu.rusu.ibc@gmail.com; 10 Romanian Institute for Evaluation and Strategy-IRES, 400495 Cluj-Napoca, Romania; 11 Department of Medical Psychology, Iuliu Haṭieganu University of Medicine and Pharmacy, 400006 Cluj-Napoca, Romania; hcoman@umfcluj.ro; 12 Department of Legal Medicine, Iuliu Haṭieganu University of Medicine and Pharmacy, 400006 Cluj-Napoca, Romania

**Keywords:** suicidal behavior, single nucleotide polymorphisms (SNPs), psychosocial determinants, biomarkers, suicide prevention

## Abstract

Suicide, a major contributor to global mortality rates, especially among young patients, remains insufficiently integrated into public health initiatives despite notable progress in identifying its determinants. The prediction of suicidal behavior remains complex, often relying on subjective assessments rather than objective biomarkers. Single nucleotide polymorphisms (SNPs) such as rs110402 (*CRHR1* gene), rs3800373 (*FKBP5* gene), and rs2289656 (*NTRK2* gene) have been linked to physiological mechanisms involving stress response and activation of the hypothalamic–pituitary–adrenal (HPA) axis, which contributes to anxiety and stress regulation. This study aimed to assess stress-related gene polymorphisms in individuals with suicidal behavior compared to controls. According to our results, the presence of the A allele of rs2289656 was associated with a protective effect, while the GG genotype conferred a higher susceptibility to suicidal behaviors. Significant associations were observed between trauma and abuse history and the rs110402 polymorphism in *CRHR1* gene, highlighting a protective role for the GG genotype and increased predisposition to stress-related psychiatric conditions and suicidal behavior for A allele carriers. No valid associations were found for rs3800373 in the *FKBP5* gene, although suggestive trends related to depression and self-aggression were noted. Our findings underscore the need to identify reliable biomarkers associated with suicide risk, highlighting the importance of integrating hereditary and psychosocial data to better understand the underlying mechanisms and to support the development of effective early interventions.

## 1. Introduction

Suicide is an under-recognized public health problem affecting all age groups worldwide [1]. In spite of the impressive level of knowledge currently available, the actual impact of the factors that trigger suicide behaviors is not elucidated to an extent allowing the identification of persons presenting an imminent risk [2], which hinders the implementation of effective preventive measures in accordance with the goals of sustainable development set by the World Health Organization [3] in view of the 2030 Agenda of the United Nations [4]

Objective indicators of biological processes, biomarkers can be quantified by observing changes in gene expression, or in the structure of various proteins, or by following the chemical changes detectable at the metabolic level in the central and peripheral nervous system [5]. Biomarkers can facilitate the diagnosis of various medical conditions, providing reliable indications in response to various treatments and suggesting new therapeutic targets [6]. However, the prediction of suicide behaviors remains difficult, as it is often based on subjective reports of the patients’ ideation or action plans [7].

As the most frequent mutations in the human genome, single nucleotide polymorphisms (SNPs) are valuable markers for understanding the molecular bases of diseases, their preservation during evolution allowing the association of DNA sequence changes with phenotypic changes [8]. A polymorphic gene has at least two alleles with a frequency greater than 1%, the one with a lower frequency being called a minor allele. The allelic frequency may vary significantly from one population to another. SNPs can be classified by frequency or by their location relative to the coding regions, given that most SNPs occur in coding regions. As gene expressions are controlled by non-coding DNA, SNPs in this region are more likely to be pathogenic [9]. It is documented that patients with mental illnesses are at higher risk of suicide attempts during their lifetime, one study finding that those with mood disorders were 8 to 30 times more likely to develop such behaviors [10].

Numerous studies have associated abnormalities in the functioning of the hypothalamic–pituitary–adrenal (HPA) axis with mental disorders, correlating in this context, the involvement of the *CRHR1* and *FKBP5* genes in inducing suicide behaviors [11].

The corticotropin releasing hormone receptor 1 (*CRHR1*) binds to the corticotropin-releasing hormone (*CRH*), a potent mediator of endocrine, autonomic, behavioral and immune responses to stress, which is the main neuroregulatory function of the HPA axis. *CRH*’s major psychiatric influence was documented by imaging studies on patients with major depression (MDD) and suicidal behavior [12]. The *CRHR1* gene (locus 17q21.31) is a key component of the serotonin circuit, encoding a protein essential for the activation of signal transduction pathways that regulate physiological processes such as stress, reproduction, immune response and obesity (National Center for Biotechnology) [13]. The FK506-binding protein 5 (*FKBP5*), a member of the immunophilin-binding protein family, is encoded by the *FKBP5* gene (locus 6p21.31). It has been shown that *FKBP5* plays an important role in stress endocrinology and in signaling through the glucocorticoid receptors, being associated with chronic pain and obesity [14]. A study found that the association of the *CRHR1* and *FKBP5* genes with trauma and adverse events in childhood is likely to lead to suicide behaviors in adulthood [15]. Other studies have correlated childhood trauma with suicide attempts, depression, neuroticism or alcohol abuse, moderated by the rs110402 and rs7209436 polymorphisms of the *CRHR1* gene [16,17,18]. A meta-analysis concluded that the rs6295 polymorphism of the *HTR1* gene may increase the risk of developing suicide behaviors [19].

Similarly, variants of the *FKBP5* gene have been associated with an increased frequency of depressive episodes [20] and suicide attempts [21,22]. A meta-analysis associated the presence of the C (rs3800373) and T (rs4713916) risk alleles in the *FKBP5* gene with a higher frequency of depressive disorders, observing that the rs1360780 and rs3800373 polymorphisms of the *FKBP5* gene are involved in the development of suicide behaviors or in cases of completed suicide [19]. The same mutations were associated with completed suicide in a Japanese population [23]. Analyzing polymorphisms in the *FKBP5* gene, another study found that the CC genotype of the rs3800373 variant is more frequent in patients with severe depression and psychosis [24]. A recent study concluded that the association of the rs3800373 variant of the *FKBP5* gene with life in large urban agglomerations influences neuronal activity in brain areas sensitive to stress [25]. As in the case of the *CRHR1* gene, it has been shown that childhood trauma exposes individuals possessing rs3800373 to an increased risk of developing mental illnesses in adulthood [26].

Associated with various mental illnesses, neurotrophins are the most studied subgroup of neurotrophic factors, a family including the nerve growth factor NGF, the brain-derived neurotrophic factor (BDNF), neurotrophin 3 (NT3) and neurotrophin 4 (NT4). They play an important role in the central and peripheral nervous system, modulating the survival, development, function and plasticity of neurons. BDNF (brain-derived neurotrophic factor) has diverse biological functions that are mediated by the activation of two main classes of receptors, tropomyosin-related kinase (TrkB) and p75 neurotrophin receptor (p75NTR). Dysregulation of BDNF signaling cascades has been suggested to underlie the pathogenesis of some common and rare diseases [27].

The BDNF factor is involved in brain aging and in the pathophysiology of Alzheimer’s, Parkinson’s and Huntington’s diseases [28]. It binds to TrkB and the low-affinity nerve growth factor (LNGFR/p75) receptors, being able to modulate the activity of other neurotransmitters. Both BDNF and TrkB are widely expressed in the hippocampus and neocortex [29]. A member of the receptor tyrosine kinase family, TrkB is encoded by the *NTRK2* gene located on chromosome 9, locus 9q21.33. When the p75 receptor is activated, it leads to activation of the Nuclear factor-kB (NF-kB) receptor. In the absence of Trk receptors, neurotrophic signaling may trigger apoptosis rather than survival pathways. It is known that the activation of the BDNF-TrkB pathway is important in the development of short-term memory [30]. The Val66Met polymorphism of the BDNF gene is widely implicated in several psychiatric disorders, being associated with some changes in hippocampal volume with HPA axis activity, major depression, anxiety, unipolar disorder and bipolar disorder, respectively. Consequently, the genotyping of this polymorphism was used in a variety of studies in the field of psychiatric genetics [31,32]. The Val66Met polymorphism was also associated with motor recovery in multiple sclerosis [33], being also identified as the best candidate involved in the antidepressant response from a group of SNPs including rs3800373 in the *FKBP5* gene [34]. On the other hand, the CC genotype of the rs2289656 polymorphism in the *NTRK2* gene was associated with an increased risk of suicide behavior in patients with depression [35].

In this study, we aimed to investigate the potential role of specific molecular biomarkers in suicide risk by (i) analyzing the distribution of selected polymorphisms in the *CRHR1*, *FKBP5*, and *NTRK2* genes among 196 individuals with and without suicidal behaviors, (ii) assessing the association between these variants and the severity of suicidal tendencies or the presence of psychiatric disorders, and (iii) examining the interaction between molecular markers and psychosocial factors through the integration of clinical, socio-demographic, and molecular data. By targeting gene variants previously linked to suicidal behavior, we sought to identify reliable indicators of suicide risk. The polymorphisms studied included rs110402 (A;A, A;G, G;G) in *CRHR1*, rs3800373 (A;A, A;C, C;C) in *FKBP5*, and rs2289656 (A;A, A;G, G;G) in *NTRK2*.

## 2. Results

The study population included individuals from Romania (98), with more than half having secondary education (32 high school graduates, 25 vocational school graduates). The group also included 22 individuals with higher education and, at the lower end of the educational spectrum, 17 individuals with gymnasium-level education (eight years of schooling). Both the predominantly urban case group (mean age 39.69 ± 1.79 years) and the predominantly rural control group (mean age 40.88 ± 1.39 years) comprised 67 males and 31 females. Regarding marital status, 28 individuals were married, 8 were in a consensual relationship, 46 were single, 7 were divorced, 2 were separated, and 7 were widowed. In 31 cases, family members (parents or children) provided some degree of social support compensating for the absence of a partner. Religious affiliation was consistent with participants’ nationality: among the Romanian subjects, 63 were Orthodox and 1 was Greek Catholic; among the Hungarian subjects, 15 were Reformed, 2 Unitarian, and 3 Roman Catholic. Additionally, the group included 4 atheists and 10 individuals of other religious beliefs.

The socio-demographic questionnaire generated relevant results regarding psychosocial factors. Assessments of working conditions ranged from “very good” (3 respondents), “good” (24), and “satisfactory” (6), to “bad” (10) and “very bad” (4). Workplace relationships were rated as “excellent” (6 respondents), “good” (25), “acceptable” (7), “bad” (6), or “very bad” (4). Most respondents reported experiencing work-related stress (28 out of 41), while overwork was less frequently cited (only 17 out of 41 respondents). Nine subjects declared that they held leadership or coordination positions, while the rest performed execution-level jobs. Half of the respondents had changed between 1 and 5 times, while one individual reported changing jobs 45 times. Regarding previous psychiatric admissions (between 2013 and 2020), most respondents (32) reported one admission, while 12 reported two, and 9 reported three admissions. Five patients had more than 20 hospitalizations, with the highest number recorded being 77 admissions.

### 2.1. Analysis of Gene Polymorphisms and Suicide Risk

Our real-time PCR results proved particularly suitable for the identification of multiple SNPs within a small genomic region. Three SNPs were analyzed (rs110402 in the *CRHR1* gene, rs2289656 in the *NTRK2* gene, and rs3800373 in the *FKBP5* gene), with the frequencies of the relevant alleles (A/G or A/C) compared between the case and control groups.

Regarding the descriptive analysis of the sample, the Kolmogorov–Smirnov and Shapiro–Wilk normality tests (Table 1) indicated that the variable age did not follow a normal distribution (*p* = 0.014 and *p* = 0.005, respectively), while the suicide ideation score was normally distributed (*p* > 0.05 for both tests). These results imply that non-parametric statistical methods should be applied when analyzing age, whereas parametric tests are appropriate for the suicide ideation score.

Furthermore, our results revealed that the loneliness score variable shows a significant deviation from normality (*p* = 0.001). However, in contrast, the quality-of-life index did not deviate significantly from the normal (*p* = 0.200 and *p* = 0.416, respectively) (Table 1).

#### 2.1.1. CRHR1 Gene Polymorphism and Its Association with Demographic and Psychosocial Determinants

The association between SNPs and suicidal behavior was evaluated by comparing the genotypic and allelic distributions between the case and control groups. Analyses primarily focused on the rs110402 polymorphism of the *CRHR1* gene.

The distribution of genotypes and alleles (A and G; AA, AG, and GG) showed no statistically significant differences between cases and controls, as indicated by the Chi-square test (*p* > 0.05 for all comparisons). Odds ratios (OR) and relative risks (RR) for the A and G alleles, as well as for each genotype, showed no significant association with suicidal behavior, with all 95% confidence intervals including the value of 1.

**Figure 1:**
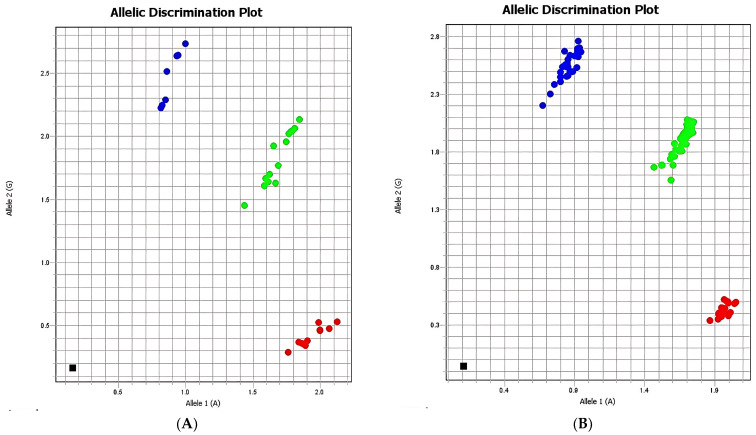
Allelic discrimination of rs110402 (*CRHR1* gene): (**A**) case group, (**B**) control group. Red: allele 1 (A)/allele 1 (A) homozygotes; blue: homozygous allele 2 (G)/allele 2 (G); green: heterozygous allele 1 (A)/allele 2 (G); black: negative control.

Specifically, the A allele showed an OR of 1.37 (95% CI: 0.72–2.59) and RR of 1.16 (95% CI: 0.87–1.56), while the G allele showed an OR of 0.70 (95% CI: 0.37–1.31) and RR of 0.83 (95% CI: 0.59–1.17). Similarly, the genotypes AA, AG, and GG did not show statistically significant differences, with *p*-values of 0.26, 0.89, and 0.33, respectively. Allelic discrimination plots confirmed a clear separation between homozygous AA, homozygous GG, heterozygous AG individuals, and negative controls (Table 2 and Figure 1).

When associations with demographic and psychosocial variables were explored, no significant associations were found with age, gender, or ethnicity overall, although a significant difference was noted for ethnicity regarding the AA genotype (*p* = 0.018), with a higher prevalence among Romanian participants compared to Hungarian participants. Regarding psychosocial determinants, two significant associations were identified. Furthermore, exposure to trauma and abuse was significantly associated with the presence of the A allele (*p* = 0.029), suggesting that individuals carrying this allele who experienced traumatic events may have an increased inherited suicidal predisposition. On the other hand, exposure to abuse showed a significant yet protective association (*p* = 0.046) for the GG genotype, indicating a complex interplay between inherited predispositions and environmental influences (Table 3).

Other psychosocial variables, including somatic and psychiatric pathology, family history of hereditary diseases, chronic diseases, experiences of deception or disillusion, depression, self-aggression, alcohol consumption, drug addiction, obsessive ideas, and memory disturbances, did not show statistically significant associations with rs110402 alleles or genotypes (Table 4).

#### 2.1.2. FKBP5 Gene Polymorphisms and Association with Demographic and Psychosocial Determinants

The analysis of the rs3800373 polymorphism in the *FKBP5* gene involved a comparison of allele and genotype frequencies between case and control groups, as well as the assessment of their association with demographic, clinical, and psychosocial factors.

Odds ratio (OR) and relative risk (RR) analyses showed that none of the evaluated alleles or genotypes (A, C, AA, AC, CC) had a statistically significant association with suicidal behaviors (Table 5).

**Figure 2:**
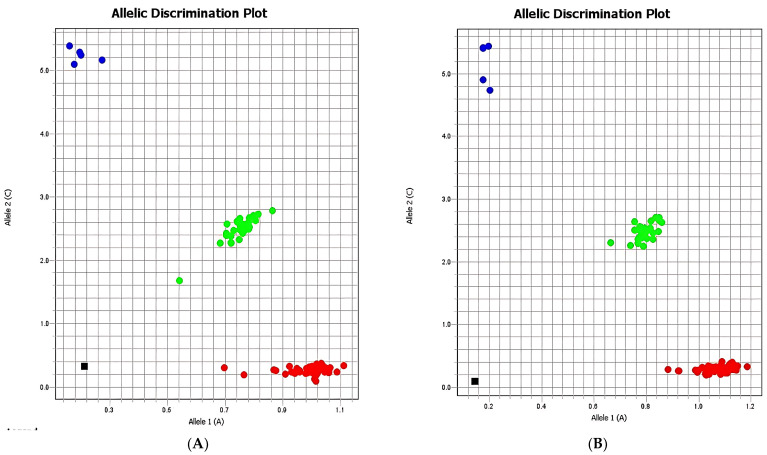
Allelic discrimination of rs3800373 in the *FKBP5* gene ((**A**): case group, (**B**): control group). Red: homozygotes for allele 1 (A/A); blue: homozygotes for allele 2 (G/G); green: heterozygotes (A/G); black: negative control.

The allelic discrimination illustrated in Figure 2 confirms the distribution of genotypes within the case and control groups, where homozygous A/A individuals are marked in red, homozygous G/G individuals in blue, heterozygous A/G individuals in green, and negative controls in black. No significant differences in genotype frequencies between groups were detected, suggesting that the general distribution of rs3800373 variants does not differ markedly between subjects with or without suicidal behavior.

The analysis of demographic determinants revealed no significant associations between rs3800373 variants and age, gender, or ethnicity, indicating a homogeneous distribution of genotypes across these factors (Table 6). Regarding clinical and psychosocial determinants, significant results were obtained: the presence of somatic pathology was significantly associated with the C allele (*p* = 0.016), suggesting that carriers may be predisposed to somatic comorbidities. No significant associations were found between the rs3800373 variants and psychiatric pathology, trauma history, abuse, chronic conditions, death of close relatives, deception, substance use, obsessive ideas, or memory dysfunction (Table 6).

Despite the lack of significance in the OR/RR estimates, additional analyses identified statistically significant associations between the A allele and self-aggressiveness (*p* = 0.042), as well as between the CC genotype and self-aggressiveness, although these results were obtained under conditions where more than 20% of the expected frequencies were below five, suggesting caution in interpreting these findings. Similarly, a trend toward an association with depression was noted for both the C allele (*p* = 0.070) and the AA genotype (*p* = 0.070), with statistical significance being reached in carriers of the AC genotype (*p* = 0.022), thereby supporting a potential involvement of *FKBP5* polymorphisms in the pathophysiology of depressive symptoms (Table 7).

#### 2.1.3. *NTRK2* Gene Polymorphisms and Its Associations with Demographic and Psychosocial Determinants

The odds ratio (OR) and relative risk (RR) analysis of developing suicidal behaviors for subjects possessing the analyzed SNPs indicated a statistically significant protective effect for the A allele of rs2289656 (OR = 0.54, 95% CI: 0.30–0.97, *p* = 0.04; RR = 0.74, 95% CI: 0.56–0.98). Conversely, the GG genotype was associated with an increased risk of suicide behaviors (OR = 1.86, 95% CI: 1.03–3.35; RR = 1.35, 95% CI: 1.02–1.77, *p* = 0.04), marking it as a potential susceptibility factor. No significant associations were found for the AG and AA genotypes independently (Table 8).

The analysis of SNP rs2289656 revealed a statistically significant difference in allele distribution between cases and controls. Specifically, the A allele was more prevalent among controls, whereas the G allele—and particularly the GG genotype—was more frequent among cases, indicating a possible risk-related genetic profile. However, no statistically significant associations were observed with demographic variables (age, sex, ethnicity), psychosocial factors (trauma, abuse), or pathological conditions (somatic, psychiatric, hereditary antecedents), which implies that the observed genetic effect may act independently of these determinants (Table 9).

Statistically significant associations were identified between the rs2289656 polymorphism of the *NTRK2* gene and deception/disillusion. Specifically, the presence of the A allele (*p* = 0.012), the AG genotype (*p* = 0.042), and the GG genotype (*p* = 0.012) were significantly associated with deception/disillusion, suggesting a possible link between this SNP and specific psychosocial traits (Table 10).

Regarding psychosocial determinants no significant associations were observed for depression, self-aggression, trauma, abuse, psychiatric or somatic pathology, chronic conditions, death of close relatives, alcohol consumption, narcomania, obsessive ideas, or memory dysfunction (Table 10).

**Figure 3:**
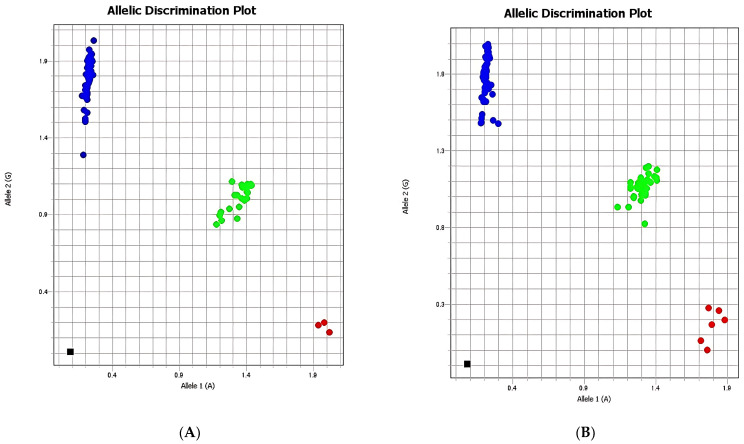
Allelic discrimination of rs2289656 (*NTRK2*): (**A**) case group, (**B**) control group. Red: allele 1 (A)/allele 1 (A) homozygotes; blue: homozygous allele 2 (G)/allele 2 (G) homozygotes; green: heterozygous allele 1 (A)/allele 2 (G); black: negative control.

The allelic discrimination analysis illustrated in Figure 3 demonstrates the distribution of rs2289656 genotypes between the case and control groups, showing a higher frequency of the G allele and the GG genotype among individuals with suicidal behaviors. Comparisons of rs2289656 distribution across demographic determinants revealed no significant differences related to age, gender, or ethnicity.

In summary, while the A allele of rs2289656 appears to exert a protective effect against suicidal behaviors, the GG genotype may be associated with an increased predisposition to such behaviors. Furthermore, its correlation with experiences of deception or disillusion suggests a potential psychosocial pathway through which this variant may influence predisposition to stress-related psychiatric conditions and suicidality. These findings indicate that the rs2289656 polymorphism of the *NTRK2* gene may modulate suicide-related behavioral outcomes and psychosocial adjustment, warranting further investigation in larger and more diverse cohorts.

## 3. Discussion

In recent decades, various SNPs involved in the functioning of biological systems have been identified, including those influencing mental illnesses or chronic conditions likely to trigger suicidal behaviors. Polymorphisms facilitate the understanding of the molecular bases of some diseases, allowing the association of changes in DNA sequences with certain phenotypic changes. Regarding the neurobiological basis of suicidal behavior, there is ample evidence that it is related to altered HPA axis and stress response [35,36,37,38].

In the context of analyzing the association between genetic variations and suicidal behaviors, these findings highlight the necessity of considering age as a potential confounding factor, given its significant influence on suicide ideation scores. Therefore, the interpretation of differences in allelic frequencies between cases and controls must include statistical adjustments for age to ensure the validity of conclusions regarding the independent genetic effects. Numerous studies have reported a heritability estimate of up to 50%, but to date, meta-analyses and GWAS have not yet identified a single, highly replicated variant associated with the full spectrum of suicidal behavior. This may be due to the complex phenotypic heterogeneity and the contribution of rare variants or gene and environment interactions. The concept should be understood as a biologically based predisposition that increases the likelihood of suicidal behavior when interacting with environmental or psychosocial stressors. Thus, integrating psychosocial assessment with molecular analysis is crucial for a comprehensive understanding of suicide risk [39].

A multitude of studies have associated this behavior with the deregulation of some biological processes, the serotonergic, noradrenergic, dopaminergic or glutamatergic dysfunctions of the HPA axis being often invoked in this context [40,41,42].

Similar associations were also found for the neurotrophic factors [30,31,32,43,44]. From this perspective, this study focused on identifying specific polymorphisms observed in patients with suicide attempts admitted to the Psychiatry Clinic of SCJU Cluj or in the samples collected from the suicides autopsied at the Cluj County Institute of Legal Medicine.

Examining the influence of rs110402 on brain responses to negative emotions under the influence of alcohol consumption in adolescents and young adults at high risk for alcoholism, a study demonstrated by functional magnetic resonance imaging (MRI) that a region of the right ventrolateral prefrontal cortex was significantly more involved in G homozygotes than in A allele carriers [45]. Another study provided psycho-behavioral and neuroendocrine evidence indicating a predisposition to stress reactivity and emotional dysregulation in homozygous individuals with the GG genotype, based on the investigation of rs110402’s role in the brain’s response to emotional stimuli [46].

Using a binary logistic regression model, a pilot study analyzing gene–environment interactions in affective disorders found statistically significant results for rs110402 polymorphisms, reflecting the impact of childhood trauma on suicide attempts [47]. Another study found that genotypic variants of rs110402 moderate the association of childhood maltreatment with neuroticism [17].

Analyzing 12 SNPs of the *CRHR2* and *CRHR1* genes, a Japanese study revealed that the T/A haplotype of rs7209436-rs110402 is significantly associated with major depression [48]. The rs110402 variant was associated with a protective role against the onset of depression in adults maltreated in childhood [16,49]. More specifically, the A allele of rs110402 helps prevent the development of depression in adult males with childhood traumas [50]. However, other researchers have associated rs110402 with an increased risk of having a first depressive episode at a young age [51].

We found that the A allele of rs2289656 exerts a protective effect against the development of suicidal behaviors, while the G/G genotype acts as a susceptibility factor for its development.

Increased risk rates were also observed for the A (rs110402-*CRHR1* gene), C (rs3800373-*FKBP5* gene) and G (rs2289656-*NTRK2* gene) alleles, respectively, as well as the AA (rs110402-*CRHR1* gene) and AC genotypes (rs3800373-*FKBP5* gene), but these results were not statistically significant.

Statistically significant associations (protective roles) were found for trauma (*p* = 0.029) and abuse (*p* = 0.046) in the suicidal patients having the GG genotype of rs110402. The probability of having suffered trauma or abuse was higher in the case of patients possessing the A allele of rs110402 (Table 3).

The *FKBP5* gene has been associated with high rates of depression [52] or other mental illnesses [20]. The CC genotype of rs3800373 was identified with higher frequency in HIV-infected patients with major depression, [24] and it was determined that this SNP also negatively affects elderly patients under antidepressant medication [53].

Associations between environmental factors and depression [54] or post-traumatic stress have been found in adults having experienced maltreatment or stressful events in childhood. Four SNPs of *FKBP5* (rs9296158, rs3800373, rs1360780 and rs9470080) were correlated with severity of childhood abuse and prediction of post-traumatic stress symptoms in adulthood [16]. Assuming that aggressive behaviors induced by childhood trauma are associated with the dysregulation of the HPA axis, other researchers analyzed the four SNPs in prisoners with aggressive behaviors and found an overexpression of the *FKBP5* gene in subjects exposed to childhood trauma, particularly in those with a history of physical abuse [55].

Various studies [15,22] have highlighted associations between *FKBP5* gene polymorphisms and suicidal behaviors, the rs3800373-rs1360780 haplotypes being significantly associated with completed suicide in a Japanese population [56].

In the present study, no valid associations were identified for the *FKBP5* rs3800373 polymorphism. A susceptibility role for the holders of the A allele and a protective role for the holders of the CC genotype were associated with self-aggression (*p* = 0.042), but more than 20% of the values had expected frequencies below 5. A similar situation was observed in the depressed subjects with the AC genotype (*p* = 0.022).

Investigating the impact of *NTRK2* gene polymorphisms (involved in major depression), a study identified the CC, CT and TT genotypes of rs2289656 as strongly associated (*p* = 0.0008) with acute suicidal behavior, suggesting the evaluation of *NTRK2* polymorphisms as a method of predicting the phenomenon [35]. Another study found associations of five SNPs located in the *NTRK2* gene with a high risk of lifetime suicide attempts in depressed patients [57].

In our analysis of the rs2289656 polymorphism we identified two situations with a susceptibility role for the development of suicidal behaviors in association with deception/disillusion, in patients possessing the A allele (*p* = 0.012) and the AG genotypes (*p* = 0.042). In contrast, the presence of the GG genotype (*p* = 0.012) exerted a protective role.

Pharmacogenetic insights are increasingly recognized for their role in guiding antidepressant treatment and understanding suicide risk [58]. Some studies have identified significant associations of *NTRK2* gene polymorphisms with responses to lithium drug treatment in patients with bipolar disorder [59,60], Alzheimer’s disease [61] or cognitive performance in the elderly [62]. Given the associations identified in our study, particularly those involving *CRHR1*, *FKBP5*, and *NTRK2*, it is important to consider the pharmacological relevance of these findings in the context of designing or optimizing treatments for stress- and anxiety-related disorders, especially in individuals with an inherited or biologically mediated predisposition to suicidal behavior. Pharmacogenomic studies support the hypothesis that individuals carrying *CRHR1* rs110402 and *FKBP5* rs3800373 risk alleles may show altered responses to antidepressants, particularly selective serotonin reuptake inhibitors (SSRI), due to dysregulated HPA axis activity [63,64]. *CRHR1* antagonists such as antalarmin have demonstrated anxiolytic effects in preclinical models and could represent a pharmacological alternative for patients with elevated stress reactivity [65]. Regarding *NTRK2*, the rs2289656 variant has been associated with differential antidepressant response and increased suicidal risk. Patients with the GG genotype appear to exhibit greater resilience, possibly benefiting more from neurotrophin-enhancing agents such as tricyclic antidepressants or ketamine [66]. These polymorphisms are also relevant in terms of toxicological outcomes. Studies have shown that individuals carrying variants affecting the HPA axis may exhibit paradoxical reactions such as agitation or increased suicidality during the initial stages of antidepressant treatment, highlighting the importance of molecular screening prior to therapy initiation [67].

Although *HTR2C* polymorphisms and 5-HT2C receptor antagonists were not analyzed in this study, their established role in serotonergic modulation, emotional dysregulation, and suicide predisposition highlights the broader relevance of serotonergic pharmacogenomics in psychiatric risk stratification [68].

This study contributes to the growing body of evidence regarding the role of specific genetic variations—such as rs110402 (*CRHR1*), rs3800373 (*FKBP5*), and rs2289656 (*NTRK2*)—in the complex etiology of suicidal behaviors and highlights their potential as biomarkers for individual risk assessment and therapeutic targeting.

Although several statistically significant associations were observed, the results must be interpreted cautiously due to important methodological limitations. The use of a candidate gene approach, though based on biologically plausible hypotheses, has been largely surpassed in recent years by genome-wide association studies (GWAS), which offer broader, hypothesis-free insights and higher replicability. Nonetheless, candidate gene studies continue to hold relevance, particularly when they are anchored in well-established neurobiological models and designed to explore gene and environment interaction dimensions often underrepresented in GWAS due to heterogeneity and phenotypic dilution. The targeted exploration of stress-regulation pathways in this study offers focused hypotheses that can inform future large-scale investigations.

Further perspectives should include integrative approaches that combine candidate gene data with polygenic risk scores, epigenetic regulation profiles, and longitudinal clinical assessments. Small-scale, deeply phenotyped cohorts like ours may serve as valuable pilot platforms for translational studies that inform precision psychiatry models. Thus, while our findings should not be overgeneralized, they offer potential directions for replication in larger, clinically stratified samples and underscore the continued need to integrate genetic, environmental, and functional genomic data in future suicide research.

## 4. Materials and Methods

### 4.1. Selection of Patients for the Case–Control Suicide Study

The study included a total of 196 subjects, divided equally into a case group and a control group. From all participants, the following socio-demographic data were collected: age, sex, urban or rural background, and level of education.

The control (n = 98) group consisted of individuals who underwent DNA testing at the Cluj Institute of Legal Medicine and had no history of severe psychiatric disorders or suicidal behavior.

The case group (n = 98) included 70 patients admitted the Psychiatry Clinic of Cluj County Emergency Hospital (SCJU Cluj) following non-fatal suicide attempts and 28 individuals who died by suicide, as confirmed by forensic examination at the Cluj Institute of Legal Medicine.

This study was designed to evaluate the association of the rs110402 polymorphism in the *CRHR1* gene, rs2289656 in the *NTRK2* gene, and rs3800373 in the *FKBP5* gene with suicidal behavior.

Inclusion criteria targeted adults over 18 years of age, with no history of severe genetic or neurological disorders that could influence the expression of the studied genes.

Patients with severe psychiatric conditions who were unable to provide informed consent were excluded from the analysis.

### 4.2. Methodology Used to Assess Depressive Symptoms and Suicidal Behavior

For the assessment of the association between the polymorphisms of the investigated genes and the severity of depressive symptoms or suicidal risk, tests were applied exclusively to individuals included in the case study group. These participants were admitted to the Psychiatry Clinic of Cluj County Emergency Hospital (SCJU Cluj), where they underwent psychiatric evaluation and diagnosis performed by certified psychiatrists.

The evaluation process involved two standardized instruments:

- **A socio-demographic questionnaire** was designed to gather detailed information on the participants’ age, gender, education level, employment status, relationship status, family background, traumatic experiences, history of abuse, psychiatric and somatic medical history, and other relevant psychosocial variables.
- **The Columbia Suicide Severity Rating Scale (C-SSRS)** is a validated instrument widely used in psychiatric research and clinical practice [69]. It was applied to assess suicidal ideation and behavior. It is used to characterize the severity of suicide risk based on factors such as the presence of suicidal thoughts, planning, past suicide attempts, and access to means. It also explores protective factors and contextual stressors. The socio-demographic data obtained were used to analyze the association between the studied polymorphisms and psychosocial risk factors. The results from the C-SSRS were used to quantify the severity of suicidal risk and to classify patients accordingly for further statistical analysis [70].

### 4.3. Biological Sample Collection and Processing

For surviving patients and control subjects, peripheral blood samples were collected by specialized medical personnel using EDTA vacutainer tubes. In the case of individuals who died by suicide, biological samples (brain tissue or other post-mortem DNA sources) were collected and preserved according to the standard medico-legal protocol. Genomic DNA extraction and purification were performed using a commercial kit designed for DNA extraction from blood and tissue (Ready DNA Spin Kit, inno-train Diagnostic GmbH, Kronberg, Germany). The purity and concentration of the extracted DNA were determined by UV spectrophotometry using the NanoDrop 2000 system (Thermo Fisher Scientific, USA).

### 4.4. Genotyping and SNP Selection

For the determination of the genotypes of the SNPs of interest, the TaqMan^®^ SNP Genotyping Assays technology was used through real-time PCR (RT-PCR). Specific primers for each SNP were used, following the protocols provided by the manufacturer. The SNP analysis was performed using the Applied Biosystems system by Thermo Fisher Scientific Inc. (Waltham, MA, USA). For each SNP analyzed, specific primers were used, as presented in Table 11.

DNA samples were stored at −20 °C and, prior to genotyping, were brought to room temperature and homogenized. Each PCR reaction was performed in a final volume of 25 µL, containing

- 1.25 µL of 20× primer stock solution;
- 12.5 µL of 2× TaqMan^®^ Genotyping Master Mix;
- 11.25 µL of purified genomic DNA (1–20 ng/well, final concentration 0.2 ng/µL).

A negative control consisting of nuclease-free ultrapure water was included. The reaction plate was sealed with adhesive film and centrifuged to prevent the formation of air bubbles that could interfere with the PCR reaction.

### 4.5. RT-PCR Amplification Protocol

SNP amplification and detection were performed using the QuantStudio™ 5 Real-Time PCR System (Applied Biosystems, Thermo Fisher Scientific, USA). The amplification protocol included the following steps:

- Enzyme activation: 10 min at 95 °C;
- Forty PCR cycles, each consisting of DNA denaturation for 15 s at 95 °C and primer annealing and extension for 1 min at 60 °C.

Data were collected and analyzed using the HID Real-Time PCR Analysis Software v1.3 (Applied Biosystems, Foster City, CA, USA), and SNP genotypes were automatically determined based on the amplification curves.

### 4.6. Statistical Analysis

The distribution of SNP genotypes was assessed for conformity with the Hardy-Weinberg equilibrium using the Chi-square test.

Differences in genotype distribution between the case and control groups were analyzed using the Chi-square test and Fisher’s exact test where appropriate. All statistical analyses were performed using SPSS software version 26.0 (BM, Armonk, NY, USA), considering a significance threshold of *p* < 0.05.

### 4.7. Ethical Considerations

The study was approved by the Ethics Committee of the “Iuliu Hațieganu” University of Medicine and Pharmacy, Cluj-Napoca. All living participants provided written informed consent, and in the case of post-mortem samples, their use was conducted in accordance with legal regulations governing forensic research. Patient anonymity and data confidentiality were ensured throughout the entire study.

## 5. Conclusions

In the present study, the A allele of the rs2289656 SNP (*NTRK2* gene) was identified as exerting a protective effect against the development of suicidal behaviors, while the G/G genotype was found to act as a susceptibility factor. In individuals experiencing psychological distress, this particular polymorphism demonstrated contrasting associations with suicidal behavior: the presence of the A allele (*p* = 0.012) and the AG genotype (*p* = 0.042) were linked to increased susceptibility, whereas the GG genotype (*p* = 0.012) appeared to play a protective role.

This research highlighted various associations of the studied polymorphisms with determinants of suicidal behaviors, such as a history of abuse and trauma for the rs110402 polymorphism of the *CRHR1* gene, with a protective role for those with the GG genotype and a susceptibility role for those with the A allele. On the other hand, no valid associations were identified for the rs3800373 polymorphism in the *FKBP5* gene.

These findings underscore the relevance of integrating molecular and psychosocial data in suicide research and highlight the need for further studies in larger, more diverse populations to validate the observed associations and improve individualized risk assessment.

## Figures and Tables

**Table 1:** The Kolmogorov–Smirnov/Shapiro–Wilk normality test values for the descriptive variables.

Test	Kolmogorov–Smirnov		Shapiro–Wilk	
Variable	Coefficient	*p*	Coefficient	*p*
Age	0.131	0.014 *	0.938	0.005 **
Loneliness score	0.207	0.001 ***	0.925	0.001 ***
Quality of life index	0.079	0.200	0.979	0.416
Suicide ideation score	0.072	0.200	0.972	0.194

Significance levels: *** *p* < 0.001, ** *p* < 0.01 and * *p* < 0.05.

**Table 2:** Odds ratio (OR) and relative risk (RR) of developing suicide behaviors for subjects possessing the analyzed SNPs—*CRHR1* gene.

SNP	Allele	OR		RR		Pearson	Sig. 2-Tailed
		Val	CI 95%	Val	CI 95%	X^2^	*p*
rs110402	A	1.37	(0.72; 2.59)	1.16	(0.87; 1.56)	0.94	0.33
rs110402	G	0.70	(0.37; 1.31)	0.83	(0.59; 1.17)	1.27	0.26
rs110402	AA	1.44	(0.76; 2.71)	1.21	(0.86; 1.71)	1.27	0.26
rs110402	AG	0.96	(0.55; 1.68)	0.98	(0.74; 1.30)	0.02	0.89
rs110402	GG	0.73	(0.39; 1.38)	0.86	(0.64; 1.16)	0.94	0.33

**Table 3:** Associations of rs110402 (*CRHR1* gene) with demographic and psychosocial determinants.

rs110402 (*CRHR1*)	Categorical Variables		Allele		Genotype		
			A	G	AA	AG	GG
Sample	Case	N (%)	75 (52.1)	68 (47.6)	30 (56.6)	45 (49.5)	23 (44.2)
	Control	N (%)	69 (47.9)	75 (52.4)	23 (43.4)	46 (50.5)	29 (55.8)
	Pearson X^2^		0.942	1.267	1.267	0.021	0.942
	Degrades of freedom		1	1	1	1	1
	Sig. 2-tailed *p*		0.332	0.260	0.260	0.886	0.332
**Demographic determinants**							
Age	Average (years)		42.55	42.33	43.49	42.0	42.9
Gender	Female	N (%)	98 (68.1)	102 (71)	32 (60.4)	66 (72.5)	36 (69.2)
	Male	N (%)	46 (31.9)	41 (28.7)	21 (39.6)	25 (27.5)	16 (30.8)
	Pearson X^2^		0.24	2.144	2.144	0.942	0.24
	Degrades of freedom		1	1	1	1	1
	Sig. 2-tailed *p*		0.876	0.143	0.143	0.332	0.876
Ethnicity	Romanians	N (%)	53 (70.7)	56 (82.4)	16 (53.3)	37 (82.2)	19 (82.6)
	Hungarians	N (%)	19 (25.3)	11 (16.2)	12 (40)	7 (15.6)	4 (17.4)
	Rroma	N (%)	2 (2.7)	1 (1.5)	1 (3.3)	1 (2.2)	0
	Other	N (%)	1 (1.3)	0	1 (3.3)	0	0
	Pearson X^2^		1.735	10.041	10.041	3.951	1.735
	Degrades of freedom		3	3	3	3	3
	Sig. 2-tailed *p*		0.629 ^a,c^	0.018 ^a,^*^,c^	0.018 ^a,^*^,c^	0.267 ^a,c^	0.629 ^a,c^
**Psychosocial determinants**							
Somatic pathology	Presence	N (%)	5 (9.1)	7 (14.3)	0	5 (14.7)	2 (13.3)
	Absence	N (%)	50 (90.9)	42 (85.7)	21 (100)	29 (85.3)	13 (86.7)
	Pearson X^2^		0.236	3.333	3.333	1.627	0.236
	Degrades of freedom		1	1	1	1	1
	Sig. 2-tailed *p*		0.627 ^a^	0.068 ^a^	0.068 ^a^	0.202 ^a^	0.627 ^a^
Psychiatric pathology	Presence	N (%)	45 (81.8)	39 (79.6)	17 (81)	28 (82.4)	11 (73.3)
	Absence	N (%)	10 (18.2)	10 (20.4)	4 (19)	6 (17.6)	4 (26.7)
	Pearson X^2^		0.530	0.017	0.017	0.229	0.530
	Degrades of freedom		1	1	1	1	1
	Sig. 2-tailed *p*		0.466 ^a^	0.896 ^a^	0.896 ^a^	0.632	0.466 ^a^
Hereditary—collateral antecedents	Presence	N (%)	64 (85.3)	56 (82.4)	25 (83.3)	39 (86.7)	17 (73.9)
	Absence	N (%)	11 (14.7)	12 (17.6)	5 (16.7)	6 (13.3)	6 (26.1)
	Pearson X^2^		1.601	0.014	0.014	0.935	1.601
	Degrades of freedom		1	1	1	1	1
	Sig. 2-tailed *p*		0.206 ^a^	0.906	0.906	0.334	0.206 ^a^
Trauma	Presence	N (%)	39 (52)	29 (42.6)	16 (53.3)	23 (51.1)	6 (26.1)
	Absence	N (%)	36 (48)	39 (57.4)	14 (46.7)	22 (48.9)	17 (73.9)
	Pearson X^2^		4.760	0.957	0.957	0.903	4.760
	Degrades of freedom		1	1	1	1	1
	Sig. 2-tailed *p*		0.029 *	0.328	0.328	0.342	0.029 *
Abuse	Presence	N(%)	30 (40)	21 (30.9)	13 (43.3)	17 (37.8)	4 (17.4)
	Absence	N(%)	45 (60)	47 (69.1)	17 (56.7)	28 (62.2)	19 (82.6)
	Pearson X^2^		3.971	1.424	1.424	0.349	3.971
	Degrades of freedom		1	1	1	1	1
	Sig. 2-tailed *p*		0.046 *	0.233	0.233	0.555	0.046 *
Chronic diseases	Presence	N(%)	49 (65.3)	44 (64.7)	21 (70)	28 (62.2)	16 (69.6)
	Absence	N(%)	26 (34.7)	24 (35.3)	9 (30)	17 (37.8)	7 (30.4)
	Pearson X2		0.141	0.261	0.261	0.628	0.141
	Degrades of freedom		1	1	1	1	1
	Sig. 2-tailed *p*		0.707	0.609	0.609	0.428	0.707

* Significant at 0.05 level (2-tailed). ^a^ 20% of cells are expected to count less than 5. Chi-square test may not be valid. ^c^ The minimum expected frequency is less than 1.

**Table 4:** Associations of rs110402 (*CRHR1* gene) with demographic and psychosocial determinants (continued).

rs110402 (*CRHR1*)	Categorical Variables		A	G	AA	AG	GG
Death of close relatives	Presence	N (%)	38 (50.7)	41 (60.3)	13 (43.3)	25 (55.6)	16 (69.6)
	Absence	N (%)	37 (49.3)	27 (39.7)	17 (56.7)	20 (44.4)	7 (30.4)
	Pearson X^2^		2.541	2.420	2.420	0.007	2.541
	Degrades of freedom		1	1	1	1	1
	Sig. 2-tailed *p*		0.111	0.120	0.120	0.934	0.111
Deception/Disillusion	Presence	N (%)	48 (64)	41 (60.3)	18 (60)	30 (66.7)	11 (47.8)
	Absence	N (%)	27 (36)	27 (39.7)	12 (40)	15 (33.3)	12 (52.2)
	Pearson X^2^		1.922	0.001	0.001	1.450	1.922
	Degrades of freedom		1	1	1	1	1
	Sig. 2-tailed *p*		0.166	0.978	0.978	0.228	0.166
Depression	Presence	N (%)	69 (92)	61 (89.7)	29 (96.7)	40 (88.9)	21 (91.3)
	Absence	N (%)	6 (8)	7 (10.3)	1 (3.3)	5 (11.1)	2 (8.7)
	Pearson X^2^		0.011	1.345	1.345	0.964	0.011
	Degrades of freedom		1	1	1	1	1
	Sig. 2-tailed *p*		0.915 ^a^	0.246 ^a^	0.246 ^a^	0.326 ^a^	0.915 ^a^
Self-agression	Presence	N (%)	31 (41.3)	31 (45.6)	12 (40)	19 (42.2)	12 (52.2)
	Absence	N (%)	44 (58.7)	37 (54.4)	18 (60)	26 (57.8)	11 (47.8)
	Pearson X^2^		0.840	0.264	0.264	0.093	0.840
	Degrades of freedom		1	1	1	1	1
	Sig. 2-tailed *p*		0.359	0.607	0.607	0.761	0.359
Alcohol consumption	Presence	N (%)	34 (45.3)	30 (44.1)	13 (43.3)	21 (46.7)	9 (39.1)
	Absence	N (%)	41 (54.7)	38 (55.9)	17 (56.7)	24 (53.3)	14 (60.9)
	Pearson X^2^		0.275	0.005	0.005	0.263	0.275
	Degrades of freedom		1	1	1	1	1
	Sig. 2-tailed *p*		0.600	0.943	0.943	0.608	0.600
Narcomania	Presence	N (%)	26 (34.7)	21 (30.9)	11 (36.7)	15 (33.3)	6 (26.1)
	Absence	N (%)	49 (65.3)	47 (69.1)	19 (63.3)	30 (66.7)	17 (73.9)
	Pearson X^2^		0.589	0.317	0.317	0.018	0.589
	Degrades of freedom		1	1	1	1	1
	Sig. 2-tailed *p*		0.443	0.574	0.574	0.895	0.443
Obsessive Ideas	Presence	N (%)	61 (81.3)	52 (76.5)	24 (80)	37 (82.2)	15 (65.2)
	Absence	N (%)	14 (18.7)	16 (23.5)	6 (20)	8 (17.8)	8 (34.8)
	Pearson X^2^		2.626	0.149	0.149	1.043	2.626
	Degrades of freedom		1	1	1	1	1
	Sig. 2-tailed *p*		0.105	0.700	0.700	0.307	0.105
Memory disfunction/disruptions	Presence	N (%)	52 (69.3)	47 (69.1)	20 (66.7)	32 (71.1)	15 (65.2)
	Absence	N (%)	23 (30.7)	21 (30.9)	10 (33.3)	13 (28.9)	8 (34.8)
	Pearson X^2^		0.138	0.058	0.058	0.290	0.138
	Degrades of freedom		1	1	1	1	1
	Sig. 2-tailed *p*		0.710	0.810	0.810	0.590	0.710

^a^ 20% of cells are expected to count less than 5. Chi-square test may not be valid.

**Table 5:** Odds ratio (OR) and relative risk (RR) of developing suicide behaviors for subjects possessing the analyzed SNPs—*FKBP5* gene.

SNP	Allele	OR		RR		Pearson	Sig. 2-Tailed
		Val	CI 95%	Val	CI 95%	X^2^	*p*
rs3800373	A	1.00	(0.28; 3.57)	1.00	(0.53; 1.89)	0.00	1.00
rs3800373	C	1.55	(0.86; 2.77)	1.25	(0.92; 1.70)	2.17	0.14
rs3800373	AA	0.65	(0.36; 1.16)	0.80	(0.59; 1.09)	2.17	0.14
rs3800373	AC	1.60	(0.87; 2.91)	1.28	(0.92; 1.77)	2.32	0.13
rs3800373	CC	1.00	(0.28; 3.57)	1.00	(0.53; 1.89)	0.00	1.00

**Table 6:** Associations of rs3800373 (*FKBP5* gene) with the demographic and psychosocial determinants.

rs3800373	Categorical Variables		Allele		Genotype		
			A	C	AA	AC	CC
Sample	Case	N (%)	93 (50)	42 (56.8)	56 (45.9)	37 (57.8)	5 (50)
	Control	N (%)	93 (50)	32 (43.2)	66 (54.1)	27 (42.2)	5 (50)
	Pearson X^2^		0.000	2.171	2.171	2.320	0.000
	Degrades of freedom		1	1	1	1	1
	Sig. 2-tailed *p*		1.000 ^a^	0.141	0.141	0.128	1.000 ^a^
**Demographic determinants**							
Age	Average (years)		42.20	44.88	41.29	43.94	50.90
Gender	Female	N (%)	59 (31.7)	24 (32.4)	38 (31.1)	21 (32.8)	3 (30)
	Male	N (%)	127 (68)	50 (67.6)	84 (68.9)	43 (67.2)	7 (70)
	Pearson X^2^		0.013	0.035	0.035	0.061	0.013
	Degrades of freedom		1	1	1	1	1
	Sig. 2-tailed *p*		0.909 ^a^	0.851	0.851	0.805	0.909 ^a^
Ethnicity	Romanian	N (%)	69 (74.2)	30 (71.4)	42 (75)	27 (73)	3 (60)
	Hungarian	N (%)	21 (22.6)	12 (28.6)	11 (19.6)	10 (27)	2 (40)
	Roma	N (%)	2 (2.2)	0	2 (3.6)	0	0
	Other	N (%)	1 (1.1)	0	1 (1.8)	0	0
	Pearson X^2^		0.905	3.107	3.107	2.142	0.905
	Degrades of freedom		3	3	3	3	3
	Sig. 2-tailed *p*		0.824 ^a,c^	0.375 ^a,c^	0.375 ^a,c^	0.543 ^a,c^	0.824 ^a,c^
**Psychosocial Determinants**							
Somatic pathology	Presence	N (%)	6 (9)	6 (20)	1 (2.5)	5 (18.5)	1 (33,3)
	Absence	N (%)	61 (91)	24 (80)	39 (97.5)	22 (81.5)	2 (66,7)
	Pearson X^2^		1.896	5.833	5.833	3.544	1.896
	Degrades of freedom		1	1	1	1	1
	Sig. 2-tailed *p*		0.169 ^a,c^	0.016 ^a,^*	0.016 ^a,^*	0.060 ^a^	0.169 ^a,c^
Psychiatric pathology	Presence	N (%)	54 (80.6)	25 (83.3)	31 (77.5)	23 (85.2)	2 (66,7)
	Absence	N (%)	13 (19.4)	5 (16.7)	9 (22.5)	4 (14.8)	1 (33,3)
	Pearson X^2^		0.348	0.365	0.365	0.739	0.348
	Degrades of freedom		1	1	1	1	1
	Sig. 2-tailed *p*		0.555 ^a,c^	0.546	0.546	0.390	0.555 ^a,c^
Hereditary—collateral antecedents	Presence	N (%)	77 (82.8)	35 (83.3)	46 (82.1)	31 (83.8)	4 (80)
	Absence	N (%)	16 (17.2)	7 (16.7)	10 (17.9)	6 (16.2)	1 (20)
	Pearson X^2^		0.026	0.024	0.024	0.053	0.026
	Degrades of freedom		1	1	1	1	1
	Sig. 2-tailed *p*		0.872 ^a,c^	0.878	0.878	0.818	0.872 ^a,c^
Trauma	Presence	N (%)	43 (46.2)	19 (45.2)	26 (46.4)	17 (45.9)	2 (40)
	Absence	N (%)	50 (53.8)	23 (54.8)	30 (53.6)	20 (54.1)	3 (60)
	Pearson X^2^		0.074	0.014	0.014	0.000	0.074
	Degrades of freedom		1	1	1	1	1
	Sig. 2-tailed *p*		0.785 ^a^	0.907	0.907	0.997	0.785 ^a^
Abuse	Presence	N (%)	34 (36.6)	13 (31)	21 (37.5)	13 (35.1)	0
	Absence	N (%)	59 (63.4)	29 (69)	35 (62.5)	24 (64.9)	5 (100)
	Pearson X^2^		2.799	0.454	0.454	0.005	2.799
	Degrades of freedom		1	1	1	1	1
	Sig. 2-tailed *p*		0.094 ^a^	0.500	0.500	0.943	0.094 ^a^

* Significant at the 0.05 level (2-tailed). ^a^ 20% of cells are expected count less than 5. Chi-square test may not be valid. ^c^ The minimum expected frequency is less than 1.

**Table 7:** Associations of rs3800373 (*FKBP5* gene) with the demographic and psychosocial determinants (continued).

rs3800373	Categorical Variables		A	C	AA	AC	CC
Chronic conditions	Presence	N (%)	61 (65.6)	30 (71.4)	35 (62.5)	26 (70.3)	4 (80)
	Absence	N (%)	32 (34.4)	12 (28.6)	21 (37.5)	11 (29.7)	1 (20)
	Pearson X^2^		0.441	0.857	0.857	0.414	0.441
	Degrades of freedom		1	1	1	1	1
	Sig. 2-tailed *p*		0.507 ^a^	0.355	0.355	0.520	0.507 ^a^
Death of close relatives	Presence	N (%)	50 (53.8)	21 (50)	33 (58.9)	17 (45.7)	4 (80)
	Absence	N (%)	43 (46.2)	21 (50)	23 (41.1)	20 (54.1)	1 (20)
	Pearson X^2^		1.320	0.773	0.773	2.014	1.320
	Degrades of freedom		1	1	1	1	1
	Sig. 2-tailed *p*		0.251 ^a^	0.379	0.379	0.156	0.251 ^a^
Deception	Presence	N (%)	58 (62.4)	27 (64.3)	32 (57.1)	26 (70.3)	1 (20)
	Absence	N (%)	35 (37.6)	15 (35.7)	24 (42.9)	11 (29.7)	4 (80)
	Pearson X^2^		3.555	0.511	0.511	2.514	3.555
	Degrades of freedom		1	1	1	1	1
	Sig. 2-tailed *p*		0.059 ^a^	0.475	0.475	0.113	0.059 ^a^
Depression	Presence	N (%)	86 (92.5)	41 (97.6)	49 (87.5)	37 (100)	4 (80)
	Absence	N (%)	7 (7.5)	1 (2.4)	7 (12.5)	0	1 (20)
	Pearson X^2^		0.985	3.278	3.278	5.284	0.985
	Degrades of freedom		1	1	1	1	1
	Sig. 2-tailed *p*		0.321 ^a,c^	0.070 ^a^	0.070 ^a^	0.022 ^a,^*	0.321 ^a,c^
Self-agression	Presence	N (%)	43 (46.2)	20 (47.6)	23 (41.1)	20 (54.1)	0
	Absence	N (%)	50 (53.8)	22 (52.4)	33 (58.9)	17 (45.9)	5 (100)
	Pearson X^2^		4.119	0.418	0.418	2.500	4.119
	Degrades of freedom		1	1	1	1	1
	Sig. 2-tailed *p*		0.042 ^a,^*	0.518	0.518	0.114	0.042 ^a,^*
Alcohol consumption	Presence	N (%)	40 (43)	22 (52.4)	21 (37.5)	19 (51.4)	3 (60)
	Absence	N (%)	53 (57)	20 (47.6)	35 (62.5)	18 (48.6)	2 (40)
	Pearson X^2^		0.556	2.158	2.158	1.348	0.556
	Degrades of freedom		1	1	1	1	1
	Sig. 2-tailed *p*		0.456 ^a^	0.142	0.142	0.246	0.456 ^a^
Narcomania	Presence	N (%)	32 (34.4)	12 (28.6)	20 (35.7)	12 (32.4)	0
	Absence	N (%)	61 (65.6)	30 (71.4)	36 (64.3)	25 (67.6)	5 (100)
	Pearson X^2^		2.555	0.557	0.557	0.001	2.555
	Degrades of freedom		1	1	1	1	1
	Sig. 2-tailed *p*		0.110 ^a^	0.456	0.456	0.971	0.110 ^a^
Obsessive Ideas	Presence	N (%)	72 (77.4)	33 (78.6)	43 (76.8)	29 (78.4)	4 (80)
	Absence	N (%)	21 (22.6)	9 (21.4)	13 (23.2)	8 (21.6)	1 (20)
	Pearson X^2^		0.018	0.044	0.044	0.023	0.018
	Degrades of freedom		1	1	1	1	1
	Sig. 2-tailed *p*		0.893 ^a^	0.834	0.834	0.878	0.893 ^a^
Memory disfunction/disruptions	Presence	N (%)	63 (67.7)	30 (71.4)	37 (66.1)	26 (70.3)	4 (80)
	Absence	N (%)	30 (32.3)	12 (28.6)	19 (33.9)	11 (29.7)	1 (20)
	Pearson X^2^		0.330	0.318	0.318	0.100	0.330
	Degrades of freedom		1	1	1	1	1
	Sig. 2-tailed *p*		0.566 ^a^	0.573	0.573	0.752	0.566 ^a^

* Significant at the 0.05 level (2-tailed). ^a^ 20% of cells are expected count less than 5. Chi-square test *may not be valid***.** ^c^ The minimum expected frequency is less than 1.

**Table 8:** Odds ratio (OR) and relative risk (RR) of developing suicide behaviors for subjects possessing the analyzed SNPs-*NTRK2* gene.

SNP	Allele	OR		RR		Pearson	Sig. 2-Tailed
		Val	CI 95%	Val	CI 95%	X^2^	*p*
r2s289656	A	0.54	(0.30; 0.97)	0.74	(0.56; 0.98)	4.30	0.04 *
rs2289656	G	2.07	(0.50; 8.50)	1.36	(0.83; 2.2)	1.05	0.31
rs2289656	AA	0.48	(0.12; 1.99)	0.74	(0.45; 1.20)	1.05	0.31
rs2289656	AG	0.60	(0.32; 1.09)	0.78	(0.59; 1.03)	2.83	0.09
rs2289656	GG	1.86	(1.03; 3.35)	1.35	(1.02; 1.77)	4.30	0.04 *

Significance levels: * *p* < 0.05.

**Table 9:** Associations of rs2289656 (*NTRK2* gene) with the demographic and psychosocial determinants.

rs2289656	Categorical Variables				Allele		Genotype		
					A	G	AA	AG	GG
Sample	Case	N (%)			29 (40.3)	95 (50.8)	3 (33.3)	26 (41.3)	69 (55.6)
	Control	N (%)			43 (59.7)	92 (49.2)	6 (66.7)	37 (58.7)	55(44.4)
	Pearson X^2^				4.303	1.048	1.048	2.830	4.303
	Degrades of freedom				1	1	1	1	1
	Sig. 2-tailed *p*				0.038 *	0.306 ^a^	0.306 ^a^	0.092	0.038 *
**Demographic determinants**									
Age	Average (years)				43.18	43.01	35.11	44.33	42.33
Gender	Females			N (%)	17 (23.6)	60 (32)	2 (22.2)	15 (23.8)	45 (36.3)
	Males			N (%)	55 (76.4)	127 (68)	7 (77.8)	48 (76.2)	79 (63.7)
	Pearson X^2^				3.386	0.386	0.386	2.627	3.386
	Degrades of freedom				1	1	1	1	1
	Sig. 2-tailed *p*				0.066	0.534 ^a^	0.534 ^a^	0.105	0.066
Ethnicity	Romanians			N (%)	22 (75.9)	69 (72.6)	3 (100)	19 (73.1)	50 (72.5)
	Hungarians			N (%)	6 (20.7)	23 (24.2)	0	6 (23.1)	17 (24.6)
	Rroma			N (%)	0	2 (2.1)	0	0	2 (2.9)
	Other			N (%)	1 (3.4)	1 (1.1)	0	1 (3.8)	0
	Pearson X^2^				3.388	1.118	1.118	3.495	3.388
	Degrades of freedom				3	3	3	3	3
	Sig. 2-tailed *p*				0.336 ^a,c^	0.773 ^a,c^	0.773 ^a,c^	0.321 ^a,c^	0.336 ^a,c^
**Psychosocial determinants**									
Somatic pathology	Presence		N (%)		1 (5)	7 (10.4)	0	1 (5.9)	6 (12)
	Absence		N (%)		19 (95)	60 (89.6)	3 (100)	16 (94.1)	44 (88)
	Pearson X^2^				0.512	0.778	0.348	0.348	0.423
	Degrades of freedom				1	1	1	1	1
	Sig. 2-tailed *p*				0.474 ^a^	0.378 ^a^	0.555 ^a,c^	0.555 ^a,c^	0.515 ^a^
Psychiatric pathology	Presence		N (%)		15 (75)	53 (79.1)	3 (100)	12 (70.6)	41 (82)
	Absence		N (%)		5 (25)	14 (20.9)	0	5 (29.4)	9 (18)
	Pearson X^2^				0.438	0.784	0.784	1.243	0.438
	Degrades of freedom				1	1	1	1	1
	Sig. 2-tailed *p*				0.508 ^a^	0.376 ^a,c^	0.376 ^a,c^	0.265 ^a^	0.508 ^a^
Hereditary—collateral antecedents	Presence		N (%)		22 (75.9)	78 (82.1)	3 (100)	19 (73.1)	59 (85.5)
	Absence		N (%)		7 (24.1)	17 (17.9)	0	7 (26.9)	10 (14.5)
	Pearson X^2^				1.325	0.650	0.650	2.263	1.325
	Degrades of freedom				1	1	1	1	1
	Sig. 2-tailed *p*				0.250	0.420 ^a,c^	0.420 ^a,c^	0.132 ^a^	0.250
Trauma	Presence		N (%)		10 (34.5)	45 (47.4)	0	10 (38.5)	35 (50.7)
	Absence		N (%)		19 (65.5)	50 (52.6)	3 (100)	16 (61.5)	34 (49.3)
	Pearson X^2^				2.169	2.628	2.628	0.792	2.169
	Degrades of freedom				1	1	1	1	1
	Sig. 2-tailed *p*				0.141	0.105 ^a^	0.105 ^a^	0.373	0.141
Abuse	Presence		N (%)		7 (24.1)	34 (35.8)	0	7 (26.9)	27 (39.1)
	Absence		N (%)		22 (75.9)	61 (64.2)	3 (100)	19 (73.1)	42 (60.9)
	Pearson X^2^				2.026	1.644	1.644	0.943	2.026
	Degrades of freedom				1	1	1	1	1
	Sig. 2-tailed *p*				0.155	0.200 ^a^	0.200 ^a^	0.331	0.155

* Significant at the 0.05 level (2-tailed). ^a^ 20% of cells are expected count less than 5. Chi-square test may not be valid. ^c^ The minimum expected frequency is less than 1.

**Table 10:** Associations of rs2289656 (*NTRK2* gene) with the demographic and psychosocial determinants (continued).

rs2289656	Categorical Variables		A	G	AA	AG	GG
Chronic conditions	Presence	N (%)	17 (58.6)	63 (66.3)	2 (66.7)	15 (57.7)	48 (69.6)
	Absence	N (%)	12 (41.4)	32 (33.7)	1 (33.3)	11 (42.3)	21 (30.4)
	Pearson X^2^		1.095	0.000	0.000	1.181	1.095
	Degrades of freedom		1	1	1	1	1
	Sig. 2-tailed *p*		0.295	0.990 ^a^	0.990 ^a^	0.277	0.295
Death of close relatives	Presence	N (%)	15 (51.7)	53 (55.8)	1 (33.3)	14 (53.8)	39 (56.5)
	Absence	N (%)	14 (48.3)	42 (44.2)	2 (66.7)	12 (46.2)	30 (43.5)
	Pearson X^2^		0.190	0.593	0.593	0.023	0.190
	Degrades of freedom		1	1	1	1	1
	Sig. 2-tailed *p*		0.663	0.441 ^a^	0.441 ^a^	0.881	0.663
Deception/disillusion	Presence	N (%)	23 (79.3)	56 (58.9)	3 (100)	20 (76.9)	36 (52.2)
	Absence	N (%)	6 (20.7)	39 (41.1)	0	6 (23.1)	33 (47.8)
	Pearson X^2^		6.276	2.046	2.046	4.129	6.276
	Degrades of freedom		1	1	1	1	1
	Sig. 2-tailed *p*		0.012 *	0.153 ^a^	0.153 ^a^	0.042 *	0.012 *
Depression	Presence	N (%)	27 (93.1)	87 (91.6)	3 (100)	24 (92.3)	63 (91.3)
	Absence	N (%)	2 (6.9)	8 (8.4)	0	2 (7.7)	6 (8.7)
	Pearson X^2^		0.088	0.275	0.275	0.010	0.088
	Degrades of freedom		1	1	1	1	1
	Sig. 2-tailed *p*		0.767 ^a^	0.600 ^a,c^	0.600 ^a,c^	0.918 ^a^	0.767 ^a^
Self-agression	Presence	N (%)	13 (44.8)	42 (44.2)	1 (33.3)	12 (46.2)	30 (43.5)
	Absence	N (%)	16 (55.2)	53 (55.8)	2 (66.7)	14 (53.8)	39 (56.5)
	Pearson X^2^		0.015	0.140	0.140	0.074	0.015
	Degrades of freedom		1	1	1	1	1
	Sig. 2-tailed *p*		0.902	0.709 ^a^	0.709 ^a^	0.785	0.902
Alcohol consumption	Presence	N (%)	11 (37.9)	41 (43.2)	2 (66.7)	9 (34.6)	32 (46.4)
	Absence	N (%)	18 (62.1)	54 (56.8)	1 (33.3)	17 (65.4)	37 (53.6)
	Pearson X^2^		0.591	0.653	0.653	1.233	0.591
	Degrades of freedom		1	1	1	1	1
	Sig. 2-tailed *p*		0.442	0.419 ^a^	0.419 ^a^	0.267	0.442
Narcomania	Presence	N (%)	10 (34.5)	30 (31.6)	2 (66.7)	8 (30.8)	22 (31.9)
	Absence	N (%)	19 (65.5)	65 (68.4)	1 (33.3)	18 (69.2)	47 (68.1)
	Pearson X^2^		0.063	1.628	1.628	0.057	0.063
	Degrades of freedom		1	1	1	1	1
	Sig. 2-tailed *p*		0.802	0.202 ^a,c^	0.202 ^a,c^	0.811	0.802
Obsessive Ideas	Presence	N (%)	23 (79.3)	73 (76.8)	3 (100)	20 (76.9)	53 (76.8)
	Absence	N (%)	6 (20.7)	22 (23.2)	0	6 (23.1)	16 (23.2)
	Pearson X^2^		0.073	0.896	0.896	0.008	0.073
	Degrades of freedom		1	1	1	1	1
	Sig. 2-tailed *p*		0.787	0.344 ^a,c^	0.344 ^a,c^	0.929	0.787
Memory disfunction/disruptions	Presence	N (%)	21 (72.4)	65 (68.4)	2 (66.7)	19 (73.1)	46 (66.7)
	Absence	N (%)	8 (27.6)	30 (31.6)	1 (33.3)	7 (26.9)	23 (33.3)
	Pearson X^2^		0.312	0.004	0.004	0.363	0.312
	Degrades of freedom		1	1	1	1	1
	Sig. 2-tailed *p*		0.577	0.949 ^a,c^	0.949 ^a,c^	0.547	0.577

* Significant at the 0.05 level (2-tailed). ^a^ 20% of cells have expected to count less than 5. Chi-square test may not be valid. ^c^ The minimum expected frequency is less than 1.

**Table 11:** List of analyzed SNPs and specific primer sequences.

SNP ID	Primer Sequences	Fluorophore (Alleles)	Identification Code
**rs110402**	TAAGAAGCATTTTTCTTTGCATAAC [A/G] CAACACCAGTCCTCTGTGTTTAGAA	VIC-A/FAM-G	C___2544843_10
**rs3800373**	GAAGAGCAACTATTTATTTGTCAAC [A/C] CTACAGATTTTGTTTTTAAAAATTA	VIC-A/FAM-C	C__27489960_10
**rs2289656**	ACAAGTTCCTCAGGTACAGTGAGGC [A/G] GGGAGGTGGGCTCCAGGAGGGAGCA	VIC-A/FAM-G	C__15882271_20

## Data Availability

The original contributions presented in this study are included in the article. Further inquiries can be directed to the corresponding author(s).
